
JOURNAL INFORMATION
==============================
NLM Title Abbreviation: Alcohol Res
Journal ID: Alcohol Res
NLM Journal ID: 101594475
Journal ID: ARCR

Alcohol Research : Current Reviews

ISSN: 2168-3492
EISSN: 2169-4796
Publisher: National Institute on Alcohol Abuse and Alcoholism

ARTICLE INFORMATION
==============================
PMCID: PMC3860382
Article ID: arcr-34-4-522
Article version: 1
Subjects: Stress Glossary

Stress Glossary

Print publication date: 2012
Volume: 34
Issue: 4
First page: 522
Last page: 524
Copyright year: 2012
License: Unless otherwise noted in the text, all material appearing in this journal is in the public domain and may be reproduced without permission. Citation of the source is appreciated.
License URL: https://creativecommons.org/publicdomain/mark/1.0/

Comment in: Overview: Stress and Alcohol Use Disorders Revisited 34 4 2012 386 390 Alcohol Research : Current Reviews PMC3860385 23584104
Comment in: Neural Pathways of Stress Integration 34 4 2012 441 447 Alcohol Research : Current Reviews PMC3860392 23584110
Comment in: Treatment of Alcohol Dependence With Drug Antagonists of the Stress Response 34 4 2012 516 521 Alcohol Research : Current Reviews PMC3860394 23584117
Comment in: How Does Stress Lead to Risk of Alcohol Relapse? 34 4 2012 432 440 Alcohol Research : Current Reviews PMC3788822 23584109

==============================
Adrenocorticotropic hormone (ACTH): Also known as corticotropin, is a hormone produced and secreted by the anterior pituitary gland. It is an important component of the hypothalamic–pituitary–adrenal (HPA) axis and often is produced in response to biological stress (along with corticotropin-releasing factor from the hypothalamus). Its principal effects are increased production and release of corticosteroids from the adrenal glands.

Agonist: An agent that mimics the actions or effects of another agent (e.g., a drug that mimics the effects of a neurotransmitter).

Allostasis: A principle first defined by Sterling and Eyer in 1988 to describe the dynamic operating range of most vital functions (e.g., blood pressure, glucose levels) and the notion that such set points change (i.e., increase or decrease) to new steady states upon repeated challenge. It also is sometimes defined as “maintenance of stability outside of the normal homeostatic range.”

Allostatic Load: Extrapolating from Sterling and Eyer’s (1988) concept of allostasis, McEwen and Stellar (1993, p. 2094) defined this term as the state of an organism wherein, “strain on the body produced by repeated ups and downs of physiologic response, as well as by the elevated activity of physiologic systems under challenge, and the changes in metabolism and the impact of wear and tear on a number of organs and tissues, can predispose the organism to disease.”

Allostatic State: “A state of chronic deviation of the regulatory systems from their normal state of operation with establishment of a new set point” (Koob and Le Moal 2001, p. 102). In these authors’s allostatic model of alcohol and other drug addiction, drug reward set points have been altered below the normal (homeostatic) set point.

Amygdala: Almond-shaped groups of nerve cells (i.e., neurons) located deep in the brain that are involved in the processing and memory of emotional reactions.

Antagonist: An agent that blocks or reverses the actions or effects of another agent (e.g., a drug that blocks the effects of a neurotransmitter).

Axon: The long fiber extending from the body of a nerve cell along which impulses are conducted to other cells.

Bed nucleus of the stria terminalis (BNST): One of the structures that make up the extended amygdala.

Catecholamines: A group of physiologically active substances with various roles in the functioning of the nervous system; also help regulate heart functioning.

Caudate nucleus: A cluster of nerve cells located within the basal ganglia of the brain that is involved with control of voluntary movement and is an important part of the brain’s learning and memory system.

Cortisol: A steroid hormone, more specifically a glucocorticoid, released in response to stress. Its primary functions are to increase blood sugar levels; suppress the immune system; and aid in fat, protein, and carbohydrate metabolism.

Dendritic spine: A small membranous protrusion from a neuron’s dendrite that typically receives input from a single synapse of an axon. Dendritic spines help transmit electrical signals to the neuron’s cell body.

Dendrites: The branched projections of a neuron that act to conduct the electrochemical stimulation received from other neural cells to the cell body, or soma, of the neuron from which the dendrites project.

Dopamine: Neurotransmitter that is involved, among other functions, in controlling behavior and cognition, motor activity, motivation and reward, mood, attention, and learning.

Dorsal striatum: A pair of nuclear masses that form the basal ganglia, along with the subthalamic nucleus and the substantia nigra.

Epigenetic: Heritable changes in phenotype or gene expression caused by mechanisms other than changes in the underlying DNA sequence.

GABAergic: Referring to neurons that use γ-aminobutyric acid (GABA) as a neurotransmitter.

Genome: The total genetic material of an organism or species.

Glucocorticoid: A class of steroid hormone (e.g., cortisol) produced in the outer layer (cortex) of the adrenal glands that bind to the glucocorticoid receptor, which is present in almost every vertebrate animal cell. They are involved in the metabolism of glucose, proteins, and fats and have anti-inflammatory properties.

Hippocampus: A curved ridge found within the cerebral hemisphere that functions in consolidation of new memories; also thought to play a role in alcohol withdrawal seizures.

Hypothalamic–pituitary–adrenal (HPA) axis: A tightly regulated signaling cascade involving hormones produced by the hypothalamus, pituitary gland, and adrenal glands. It constitutes a major part of the neuroendocrine system that controls reactions to stress but also regulates many body processes, including digestion, the immune system, mood and emotions, and energy storage and expenditure.

Hypothalamus: A portion of the brain that contains a number of small nuclei with a variety of functions. One of its most important functions is to link the nervous system to the hormonal (i.e., endocrine) system by releasing hormones that then act on the pituitary gland, inducing it to produce other hormones affecting a variety of organs.

Ligand: Any substance that binds to a receptor.

Limbic system: A set of brain structures, including the hippocampus, amygdala, anterior thalamic nuclei, septum, limbic cortex and fornix, that seemingly support a variety of functions, including emotion, behavior, motivation, long term memory, and olfaction.

Locus coeruleus: A cluster of nerve cells located in a part of the brainstem (i.e., the pons) that is involved with physiological responses to stress and panic.

Long-term potentiation: A long-lasting strengthening of signal transmission between two neurons that occurs in response to repeated activation of those neurons. It is one of the mechanisms contributing to synaptic plasticity and is thought to be one of the main mechanisms underlying memory and learning.

Mesolimbic dopamine system: System of interconnected brain regions consisting of the ventral tegmental area, nucleus accumbens, and components of the limbic system, such as the amygdala; is considered the brain’s reward pathway that mediates the rewarding effects associated with alcohol and other drug use as well as other experiences.

messenger RNA (mRNA): Key intermediary molecule generated during gene expression; mRNA levels for a gene are used as an indicator of how “active” the gene is (i.e., how much of the protein is produced).

Neuroadaptation: A change over time in response to a chronic environmental stimulus.

Neuropeptide: Protein-like molecule made in the brain. Neuropeptides consist of short chains of amino acids, with some functioning as neurotransmitters and some functioning as hormones.

Neuroplasticity: Ability of the nervous system to change and reorganize itself throughout life by forming new connections among nerve cells or altering the activities of existing nerve cells and connections; is the basis of the ability to learn throughout life.

Neurotrophins: A family of proteins that induce the survival, development, and function of neurons.

Neuropeptide Y: A neuropeptide that acts as a neurotransmitter or neuromodulator and has a role in regulating several behaviors including affect and eating.

Neurotransmitter: Signaling molecules produced in nerve cells (neurons) that serve to transmit signals from one neuron to another neuron or from a neuron to another type of cell (e.g., muscle cell); neurotransmitters are released from the signal-emitting neuron and bind to receptors on the surface of the signal-receiving cell.

Norepinephrine (noradrenaline): A catecholamine with multiple roles that may act as both a hormone and a neurotransmitter.

Nucleus/nuclei: In neuroanatomy, a group of specialized nerve cells in the brain or spinal cord that acts as a hub or transit point for electrical signals in a single neural subsystem.

Nucleus accumbens: A nucleus located in the forebrain that plays an important role in reward, pleasure, addiction, and fear; primarily contains neurons that secrete the inhibitory neurotransmitter γ-aminobutyric acid (GABA).

Paraventricular nucleus (PVN): A neuronal nucleus in the hypothalamus. It contains multiple subpopulations of neurons that are activated by a variety of stressful and/or physiological changes.

Pituitary gland: The master gland of the endocrine system, which is responsible for controlling the hormone that affects growth, metabolism, maturation, and reproduction.

Polymorphism: The presence of two or more alleles of a gene or other DNA sequence at a particular locus in a population.

Positron emission tomography: Imaging technique that produces a three-dimensional image based on signals emitted by a radioactive tracer molecule that is introduced into the body in a variant of a biologically active molecule.

Prefrontal cortex: The anterior part of the frontal lobes of the brain that has been implicated in planning complex cognitive behavior, personality expression, decision making, and moderating social behavior.

Raphe nuclei: A moderate-sized group of nuclei found in the brainstem that releases the neurotransmitter serotonin to the rest of the brain.

Receptor: A protein molecule located in the membrane surrounding a cell or in the cell’s interior that can bind with a signaling molecule (e.g., a hormone or neurotransmitter), thereby setting off a chain of biochemical reactions in the cell that alter the cell’s behavior in a specific manner.

Rett syndrome: A disorder of the nervous system that leads to developmental reversals, especially in the areas of expressive language and hand use.

Substantia nigra: A brain structure located in the midbrain (i.e., mesencephalon) that plays an important role in reward, addiction, and movement.

Synapse: A junction between two nerve cells, consisting of a minute gap across which signals are transmitted by diffusion of a neurotransmitter.

Synaptic plasticity: The ability of the connection, or synapse, between two neurons to change in strength in response to either use or disuse of signal transmission via this synapse. Synaptic plasticity also results from the alteration of the number of receptors located on a synapse.

Transcription: Biochemical process in which messenger RNA (mRNA) is generated based on the genetic information of the DNA.

Ventral striatum: A portion of the striatum. It consists of the nucleus accumbens and the olfactory tubercle.

Ventral tegmental area: Area located in the midbrain that contains dopaminergic nerve cell bodies which project to various parts of the forebrain, including the nucleus accumbens and other parts of the mesolimbic dopamine system.

